# Effect of Mesenchymal Stem Cells on Doxorubicin-Induced Fibrosis

**Published:** 2012-08-31

**Authors:** Simin Mohammadi Gorji, Abbas Ali Karimpor Malekshah, Mohammad Baghere Hashemi-Soteh, Alireza Rafiei, Kazem Parivar, Nasser Aghdami

**Affiliations:** 1. Department of Biology, Islamic Azad University, Science and Research Branch, Tehran, Iran; 2. Department of Anatomy and Embryology, Cellular and Molecular Research Center, Faculty of Medicine, Mazandaran University of Medical Science, Sari, Iran; 3. Department of Biochemistry and Genetics, Cellular and Molecular Research Center, Faculty of Medicine, Mazandaran University of Medical Sciences, Sari, Iran; 4. Department of Immunology, Cellular and Molecular Research Center, Faculty of Medicine, Mazandaran University of Medical Sciences, Sari, Iran; 5. Department of Regenerative Medicine and Cell Therapy, Cell Science Research Center, Royan Institute for Stem Cell Biology and Technology, ACECR, Tehran, Iran; 6. Department of stem cell and Developmental biology, Cell Science Research Center, Royan Institute for Stem Cell Biology and Technology, ACECR, Tehran, Iran

**Keywords:** Mesenchymal Stem Cell, Doxorubicin, Heart, Apoptosis, Fibrosis

## Abstract

**Objective:**

The aim of this study was to test the effect of intravenous injection of mesenchymal stem cells (MSCs) on doxorubicin (DOX)-induced fibrosis in the heart. We investigated the mechanisms that possibly mediate this effect.

**Materials and Methods:**

In this experimental study, fibrosis in the myocardium of adult male Wistar rats (weights 180-200 g, 9-10 weeks of age, total n=30) was created by DOX administration. DOX (2.5 mg/kg) was administered intraperitoneally 3 times a week, for a total dose of 15 mg/kg over a period of 2 weeks. MSCs from Wistar rats were separated and cultured in Dulbecco’s modified eagle medium (DMEM). The condition medium (CM) which contained factors secreted by MSCs was also collected from MSCs cultured in serum-free DMEM. Two weeks after the first injection of DOX, MSCs, CM and standard medium (SM) were transplanted via intravenous injection. Four weeks after transplantation, histological (Masson’s trichrome staining for fibrosis detection) and molecular [real-time polymerase chain reaction (RT-PCR)] analyses were conducted. In addition, insulin-like growth factor (IGF-1) and hepatocyte growth factor (HGF) in the CM were measured with an enzyme-linked immunosorbent assay (ELISA). For immunosuppressive treatment, cyclosporine A was given (intraperitoneally, 5 mg/kg/day) starting on the day of surgery until the end of study in all groups. Fibrosis rate and relative gene expression were compared by analysis of variance (ANOVA) and post-Tukey’s test. HGF and (IGF-1 in the CM were analyzed by independent sample t test. P<0.01 was considered statistically significant.

**Results:**

Our data demonstrated that intravenously transplanted MSCs and CM significantly reduced fibrosis and significantly increased Bcl-2 expression levels in the myocardium compared to the DOX group (p<0.01). However, there was no significant difference between Bax expression levels in these groups. In addition, secretion of HGF and IGF-1 was detected in the CM (p<0.01).

**Conclusion:**

We conclude that intravenous transplantation of MSCs and CM can attenuate myocardial fibrosis and increase Bcl-2 expression. This may be mediated by paracrine signaling from MSCs via anti-fibrotic and anti-apoptotic factors such as HGF and IGF-1.

## Introduction

Cardiac fibrosis is a major cause for the progression of heart failure, and its prevention is a critical goal in the treatment of heart failure (1, 2). Doxorubicin (DOX) or adriamycin (an anthracycline antibiotic) is a useful anti-tumor agent for treating several types of solid cancers, leukemias, and lymphomas. However, dose-dependent cardiotoxicity is the restricting problem of DOX that results in irreversible heart failure (3, 4). It is reported that possible mechanisms of DOX-induced cardiac damage might increase in reactive oxygen species, which in turn causes oxidative harm to cardiac mitochondria, eventually leading to apoptosis and myocardial fibrosis (5, 6). Former studies have demonstrated that cell transplantation could attenuate cardiac fibrosis of the regionally impaired myocardium, such as those prevailing after a myocardial infarction (7, 8). Several studies have shown promising results with mesenchymal stem cell (MSCs) transplantation in animal models of ischemic and DOX-induced heart failure (9-12). However the cardio-protective effect of MSCs is related to their differentiation into reparative or replacement cell types (13-16). Several recent studies have demonstrated that other mechanisms, including the regeneration of cardiomyocytes, may contribute to improved heart function (17). Recent reports have suggested that some of these reparative effects are related to paracrine factors secreted by MSCs (17, 18). In support of this paracrine hypothesis, it has been reported that MSCs secrete anti-fibrotic and anti-apoptotic factors that could potentially repair damaged heart tissue (17). In this research, we investigate whether intravenous transplantation of MSCs can attenuate cardiac fibrosis following DOX injection in a rat model. We predict that paracrine signaling also participates in the effects observed following MSC transplantation into DOX-treated rats.

## Materials and Methods

### Animals

In this experimental study, adult male Wistar rats that weighed 180-200 g (9-10 weeks of age, n=30) were obtained from the Central Animal Laboratory, Pasteur Institute, Iran. Animals were kept in the Central Animal House of the Mazandaran University of Medical Science under a 12-hour light/12-hour dark regimen at 22-24℃, fed with commercial standard rodent chow, and allowed free access to water. All animal experimental protocols were approved by the Animal Care and Use Committee of Mazandaran University of Medical Science (Mazandaran, Iran). After a two-week acclimatization period, animals were randomly assigned either to the healthy untreated control group (n=8) or the DOX treatment group (n=22).

### Induction of doxorubicin (DOX)-induced fibrosis model

DOX-induced fibrosis was generated as described previously (19, 20). DOX (Pharmacia, USA) was briefly administered intraperitoneally 3 times a week at individual doses of 2.5 mg/kg, for a total dose of 15 mg/kg over a period of 2 weeks. The rats were observed for four weeks after the final injection.

### Evaluation of doxorubicin (DOX)-induced fibrosis model

#### Histological assessment of myocardial damage

Four weeks after the final injection of DOX, hearts (n=3) from each group were rapidly removed and ventricles dissected from the atria. The myocardium was then fixed in 10% formalin for 24 hours, embedded in paraffin cut transversely and stained with Masson’s trichrome to detect fibrosis in the cardiac muscles. The size of the fibrotic areas was quantified as follows: transverse sections were randomly obtained from three levels (basal, middle, and apical) and five randomly selected fields per section (n=15 per animal) were analyzed. Then each field was scanned with a digital image analyzer (Olympus microscope and Olysai software) and the area of the collagenous fraction was calculated as the sum of all areas that contained connective tissue divided by the total area of the image.

#### RNA isolation and reverse-transcription

To evaluate apoptotic gene expression in the myocardium, from each experimental group (n=5 per group), 50 mg of ventricular tissue were disrupted and homogenized in IAzol lysis reagent (Qiagen, Europe). Total RNA was isolated using an RNeasy Lipid Tissue Mini Kit (Qiagen, Europe) according to the manufacturer’s instructions. Sample purity was assessed by the A260/A280 nm ratio with expected values between 1.8 and 2.0. Reverse-transcription reactions were performed with 1 µg total RNA using a Quantitect Reverse Transcription Kit (Qiagen, Europe) according to the manufacturer’s instructions. The purified RNA samples were briefly incubated in gDNA wipeout buffer at 42℃ for 2 minutes to effectively remove contaminating genomic DNA. After genomic DNA elimination, RNA samples were prepared for reverse transcription by using a master mix that consisted of quantiscript reverse transcriptase, Quantiscript RT buffer, and RT primer mix. The entire reaction took place at 42℃, followed by inactivation at 95℃. Followed by cDNA amplification using polymerase chain reaction (PCR) with primers which were described earlier (Table 1).

#### Real-time polymerase chain reaction (RT-PCR) analysis

Gene expression was assessed by quantitative RT-PCR. Primers of the apoptotic genes (Bax, Bcl2) and housekeeping gene (β-actin) are listed in table 1. The PCR mix in each well included the following: 12.5 µl of QuntiFast SYBR® Green PCR Master Mix (Qiagen, Europe), 9.5 µl dH2O, 0.5 µl each of the forward and reverse primers (10 pmol/µl), and 2 µl of single strand cDNA (12.5 ng/µl) in a final reaction volume of 25 µl. The PCR run was carried out on an iCycler iQ RT-PCR System (Bio-Rad) using the following program: stage 1: 3 minutes at 95℃; stage 2: 40 cycles of 30 seconds at 95℃, 35 seconds at annealing temperature (Table 1), and 30 seconds at 72℃; and stage 3: 3 minutes at 72℃. Product specificity was confirmed in the initial experiments by 2% agarose gel electrophoresis and by melting curve analysis at the end of each PCR reaction. All samples were run in duplicate and the mean value of each duplicate was used for all further calculations. Reverse transcriptase minus samples and no template controls were run together with test samples. The level of expression of each gene was normalized by that of β-actin (reference gene). The relative expression ratios were calculated by the 2^–ΔΔCT^ (Livak) method. Output data were transferred to Microsoft Excel for analysis.

#### Mesenchymal stem cells (MSCs) isolation

Mesenchymal stem Cells were prepared according to previously described methods (21). Briefly, femurs and tibias were dissected from Wistar rats, and bone marrow cells were isolated by flushing cavities with phosphate buffered saline (PBS). Bone marrow cells were plated in Dulbecco’s modified eagle medium (DMEM, Gibco Germany) with 15% fetal bovine serum (FBS, Gibco, Germany), and 10 IU/ml penicillin/streptomycin (Gibco, Germany). The cultures were then incubated in an atmosphere of 5% CO_2_ at a temperature of 37℃. Four days after plating, non-adherent cells were removed and adherent cells were propagated for four passages prior to transplantation.

#### Identification of MSCs

Flow cytometric analysis was used to determine the phenotype of rat MSCs with respect to their surface antigens. For this purpose, cells at the fourth passage were treated with 0.25% trypsin-EDTA, harvested, and washed with PBS. Then approximately 1.5×105 cells were suspended in 100 µl of PBS and incubated with 5 µl of mouse anti-rat antibodies for CD34 (Santa Cruz) and CD90-FITC conjugated (Serotec). The solutions were centrifuged at 1200 rpm for 3 minutes, then cells were dispersed in 400 µl of PBS and the labeled cells were analyzed by flow cytometry (FACS Calibur, BD). Control groups were incubated with FITC antibodies against rat IgG1 (eBioscience). WinMDI software was used to analyze the flow cytometric results.

**Table 1:** Primer sequences, expected fragment sizes and annealing temperatures of apoptotic and housekeeping genes


Name	Primer sequences (5'-3')	Size (bp)	Annealing

Bcl2	F: GGATGACTTCTCTCGTCGCTACCGT	118	60℃
	R: CGAGTGAGGATGTGCATGAA		
Bax	F: CCAGGACGCATCCACCAAGAAGC	136	60℃
	R: TGCCACACGGAAGAAGACCTCTCG		
β-actin	F: GAACCCTAAGGCCAACCGTG	105	59℃
	R: AGGCATACAGGGACAACACAGC		


To evaluate MSC nature, isolated cells from bone marrow were differentiated into osteogenic and adipogenic cell lineages. For osteogenic differentiation, MSCs were incubated with osteogenic induction medium that consisted of DMEM, 10% FCS, 10 nm dexamethasone (Sigma, USA), 10 nm Beta-glycerophosphate (Sigma, USA), and 50 mg/ml ascorbic 2 phosphate (Sigma, USA) for 4 weeks. Calcium deposits were demonstrated by alizarin red staining (22). For adipogenic differentiation, cells were exposed to adipogenic induction medium that consisted of DMEM, 10% FCS, 50 µg/ml ascorbic 2 phosphate, 50mg/ml indomethacin (Sigma, USA), and 100 nm dexamethasone for 3 weeks. We used oil red staining to confirm adipogenic differentiation (23).

##### Preparation of MSC condition medium (CM)

To prepare MSC-CM passage four MSCs were incubated in an incubator chamber (95% O_2_, 5% CO_2_, 37℃; Leec, UK) for 24 hours in serum-free DMEM (Gibco, Germany). The medium from 10^6^ cells was collected and centrifuged at 700 g for 10 minutes and passed through a 0.3 µm filter (Millipore) before use

##### Animal groups and transplantation of MSCs, MSCs-CM, and CM

**Fig 1:**
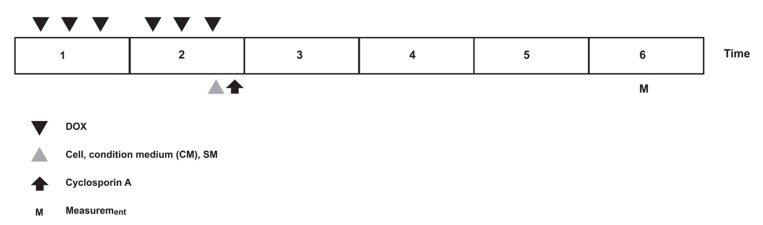
Experimental time protocols of DOX, cell, condition medium (CM) and cyclosporin A injections.

DOX-treated animals (n=22) were randomly subdivided into four groups: i.group 1 which received no further therapy (DOX group, n=5); ii.group 2 was injected with standard medium (DMEM; SM group, n=5); iii.group 3 was injected with MSCs (MSC group, n=6); and iv. group 4 was injected with MSC-CM (n=6). According to a former study that showed a modest effect with 1×10^6^ MSCs, we chose 5×10^6^ MSCs for transplantation (20). Two weeks after the first administration of DOX, we injected the following: i. 1 mL DMEM to group 2; ii. 5×10^6^ MSCs in 1 mL DMEM to group 3; and iv. 1 mL CM to group 4. Injections were performed with a 27-gauge needle and administered via the tail vein. Following injections for immunosuppressive treatment, cyclosporine A (Iran) was given (intraperitoneally, 5 mg/kg/day) starting the day of surgery and continuing until the end of study in all groups (Fig 1) (24).

### Evaluation of cell therapy

#### Histological assessment of myocardial damage after MSC and MSC-CM injections

Four weeks after MSCs, MSCs-CM, and SM injections, hearts (n=3 from each group) were rapidly removed and ventricles dissected from the atria. Transverse sections were randomly obtained from three levels (basal, middle, and apical) and five randomly selected fields per section (n=15 per animal) were analyzed. Histological assessment as mentioned above was performed.

#### RNA isolation and RT-PCR analysis

Four weeks after MSCs, MSCs-CM and SM transplantation, apoptotic gene (Bcl2 and Bax) expression in the myocardium in each experimental group (n=5 from each group) were evaluated as mentioned above.

#### Enzyme-linked immunosorbent assay (ELISA): Analysis of MSC-CMEnzyme-linked immunosorbent assay (ELISA): Analysis of MSC-CM

By measuring IGF-1 and hepatocyte growth factor (HGF) in MSC-CM, we intended to obtain information about paracrine and secretion functions of MSCs. After 24 hours incubation of MSCs (fourth passage) in serum-free culture medium, the CM was collected from 10^6^ cells and centrifuged at 10 000 g at 4℃ for 5 minutes and supernatants were stored at -20℃. Then, 100 µL of the supernatants were assayed for IGF-1 (IBL Kit, Germany; sensitivity: <5 pg/ml) and HGF (Uscn Kit, Germany; sensitivity: 6.5 pg/ml) by ELISA according to the instructions of the manufacturers.

#### Statistical analysis

Statistical analysis was performed using the Statistical Package for the Social Sciences (SPSS Version 16). Fibrosis rate and relative gene expression were compared by analysis of variance (ANOVA) and post-Tukey’s test. Secretions of HGF and IGF-1 in CM were analyzed by independent samples t-test. p<0.01 was considered statistically significant (Table 2).

## Results

### Model induction results

#### Pathological studies

**Fig 2:**
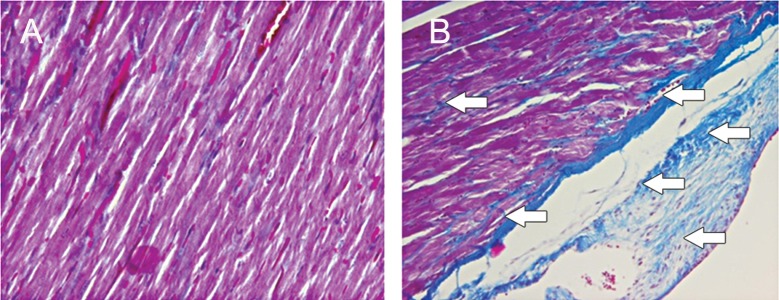
Masson’s trichrome staining; DOX significantly increased fibrosis (blue area) in the cardiac muscle compared to the control group; bar=20 µm, A. Control group; B. DOX group, (p<0.01).

**Fig 3:**
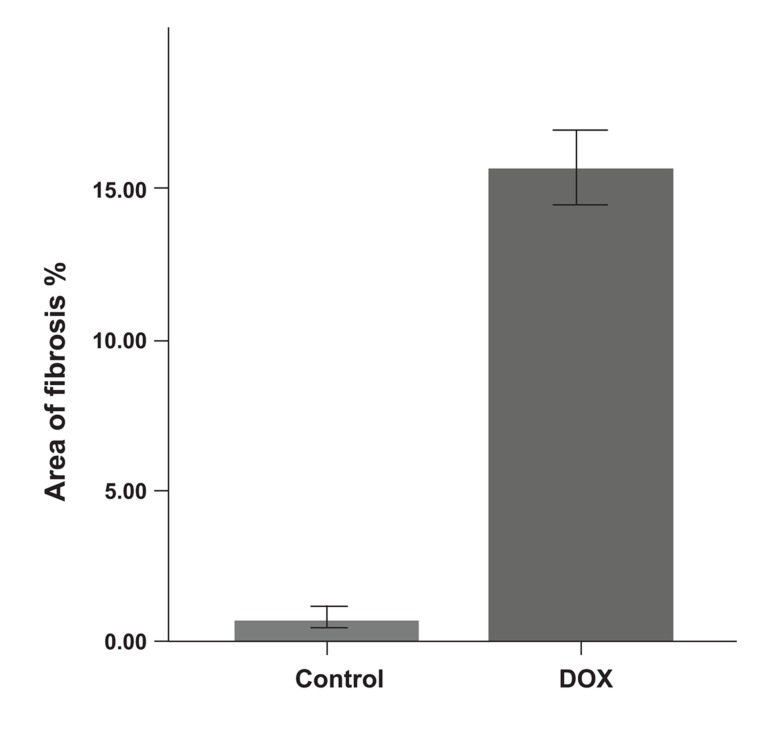
Area of fibrosis in control and DOX groups. *; p<0.01.

Histological changes of the myocardium investigated by Masson’s trichrome staining revealed that the DOX group showed fibrosis in the cardiac muscle and myocardial interstitium (Fig 2). Disproportionate accumulation of fibrosis was more concentrated near the epicardium than the endocardium. The area of interstitial fibrosis significantly increased in the DOX group (16.14 ± 1.6%) compared to the control group (1 ± 0.79%, p<0.01; Fig 3).

#### Cardiac gene expressions in the ventricles

**Fig 4:**
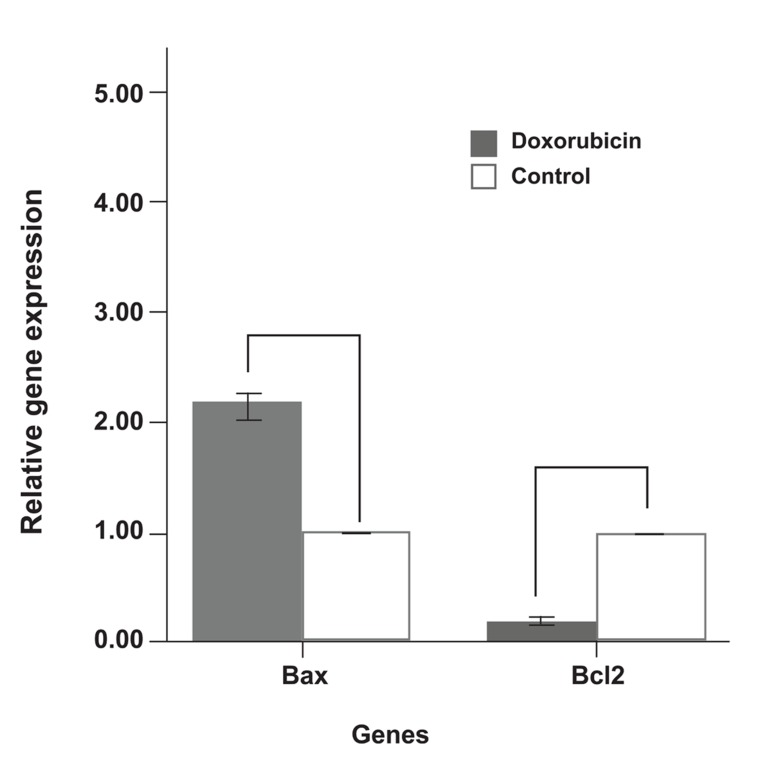
Fold differences of expression of relative apoptotic genes in DOX and control groups. *; p<0.01 .

In the DOX group levels of Bcl2 (anti-apoptotic gene) expression in the ventricles significantly decreased compared to the control group. However DOX injections significantly increased the expression levels of Bax (pro-apoptotic genes, p<0.01; Fig 4).

**Table 2:** Mean and SD of statistical analysis


		Class	N	Mean	SD	F	DF	P valua

Bc12	Control	A	5	1.0000	0.00000	69.939	4.20	0.000
	DOX	C	5	0.1856	0.07259			
	MSC	B	5	0.5700	0.10630			
	MSC-CM	B	5	0.5940	0.13686			
	SM	C	5	0.2400	0.05477			
Bax	Control	B	5	1.0000	0.00000	131.610	4.20	0.000
	DOX	A	5	2.1840	0.18730			
	MSC	A	5	2.1160	0.02608			
	MSC-CM	A	5	2.064	0.08050			
	SM	A	5	2.17	0.08367			
Fibrosis	Control	C	15	0.7034	0.78647	103.677	4.60	0.000
	DOX	A	15	15.7302	2.81805			
	MSC	B	15	10.5194	0.90676			
	MSC+CM	B	15	9.6651	1.44617			
	SM	A	15	14.8632	3.30411			

		Class	N	Mean	SD	T	DF	P valua

HGF	SM	B	5	0.0000	0.00000	-33.987	4	0.000
	MSC - CM	A	5	451.60	29.71195			
IGF	SM	B	5	0.0000	0.00000	-52.841	4	0.000
	MSC - CM	A	5	773.00	32.71085			


#### Cell therapy results

##### Characteristics of MSCs

**Fig 5:**
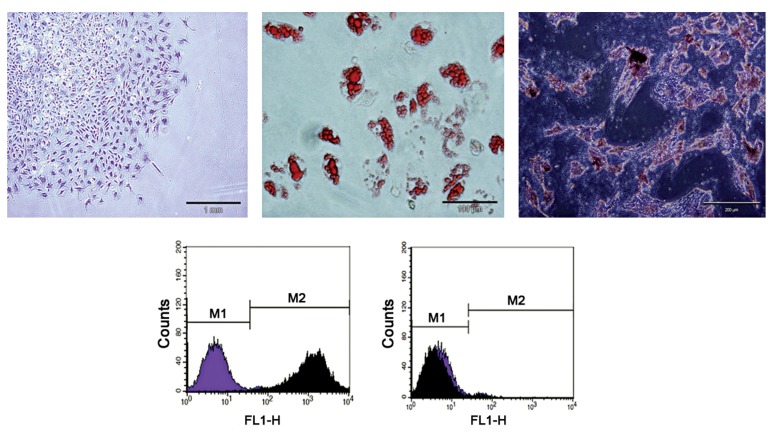
A. Adherent, spindle-shaped MSCs. B. Adipogenic induction, oil red staining. MSCs developed some lipid droplets. C. Osteogenic induction, alizarin red staining. Mineralized matrix formed in the MSCs. D. Flow cytometric analysis. Most cultured MSCs expressed CD90. In contrast, the majority of MSCs were negative for CD34.

Mesenchymal stem Cells were isolated from rat bone marrow based on adherent properties and fibroblast-like cells (Fig 5A), and characterized by flow cytometry for common stem cell surface markers (Fig 5D). The result revealed that MSCs were positive for CD90 and represented 98.55% ± 0.84% of the total cells counted. In contrast the majority of cells were negative for CD34, which represented 2.83% ± 0.46% of total cells counted. These observations were consistent with the isolation of a highly purified MSC population. In addition, the results of oil red staining and alizarin red staining also showed that MSCs had the ability to differentiate into adipocytes (Fig 5B) and osteocytes (Fig 5C).

##### Pathological studies

**Fig 6:**
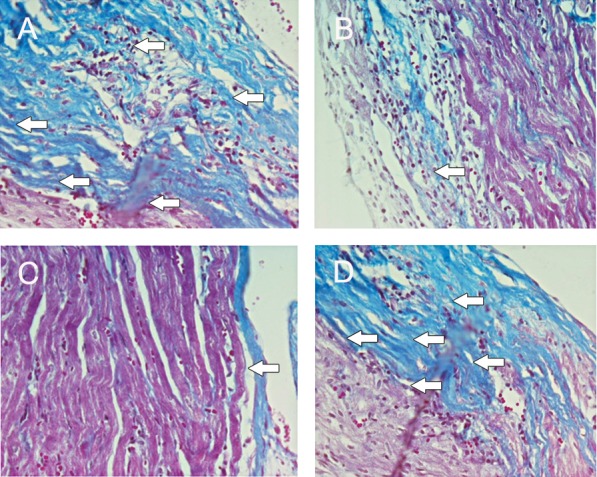
Masson’s trichrome staining. MSCs and MSCs-CM significantly decreased fibrosis (blue area) in the cardiac muscle compared to the DOX group; (bar=20 µm). A. DOX group; B. MSC group; C. MSC-CM group; D. SM group (p<0.01).

**Fig 7:**
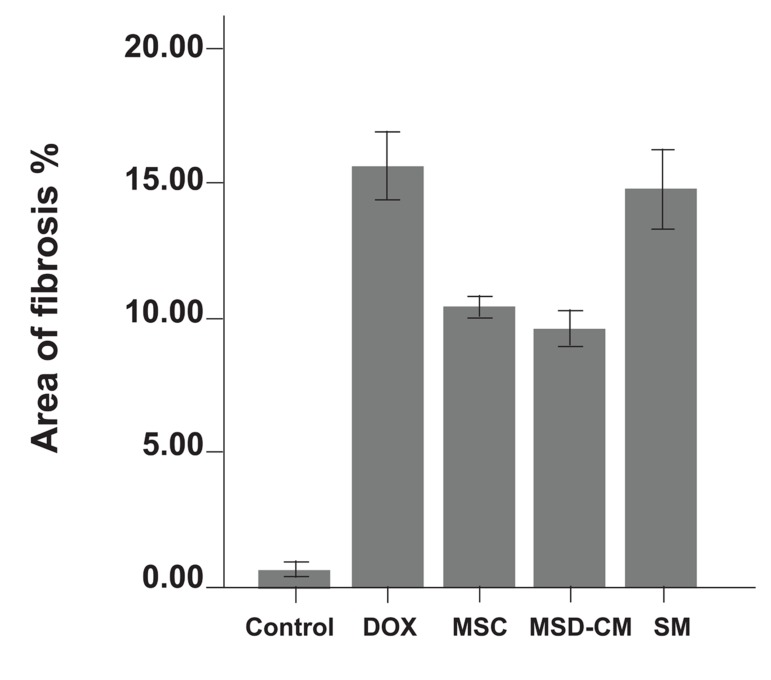
Area of fibrosis in control and experimental groups. *; p<0.01.

Histological changes of the myocardium after MSCs and MSCs-CM transplantation were investigated by staining with Masson’s trichrome. In the DOX group, atrophic cardiomyocytes were surrounded by interstitial fibrosis (16.14 ± 1.6%). MSCs and MSCs-CM exerted a protective effect on myocardium. In these groups the area of fibrosis (10.3 ± 0.9%) compared to the DOX group (8.8 ± 1.11%) was significantly reduced (p<0.01). The SM (DMEM) group showed no significant difference compared to the DOX group (Figs 6, 7).

##### Cardiac gene expressions in the ventricles

**Fig 8:**
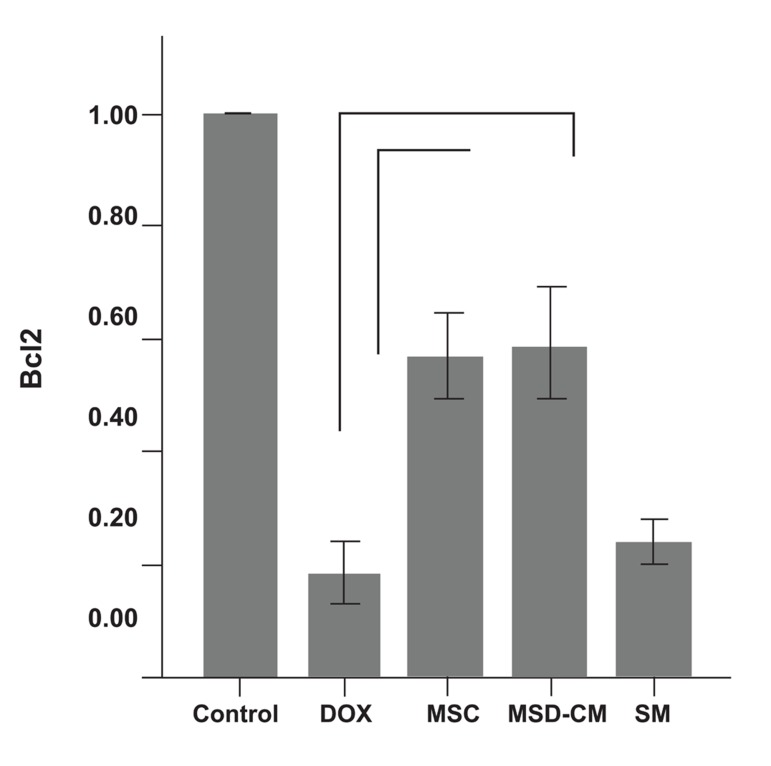
Fold differences of expression of relative Bcl2 gene in the control and experimental groups. *; p<0.01.

**Fig 9:**
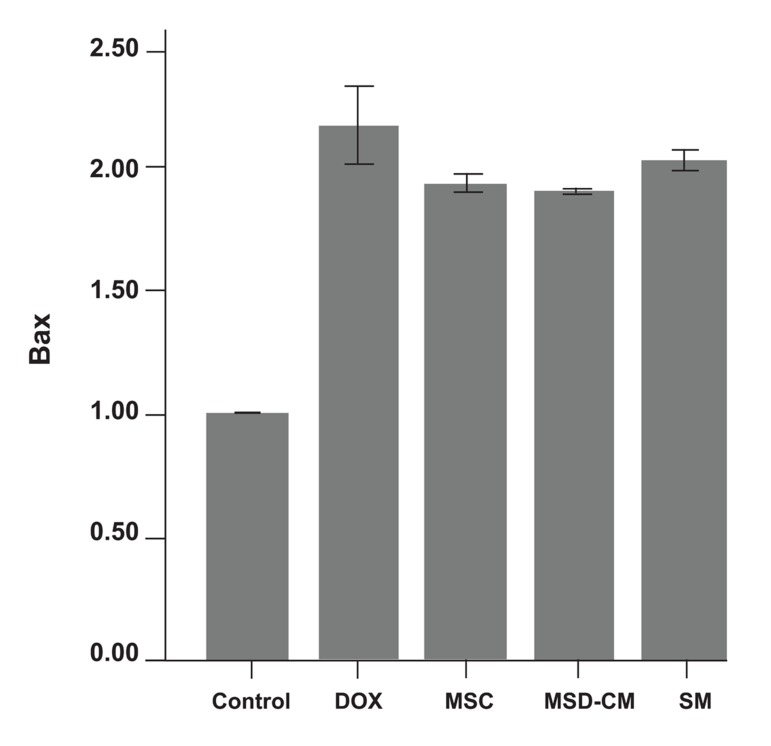
Fold differences of expression of relative Bax gene in the control and experimental groups.

Changes in the expression levels of Bcl-2 and Bax in the rat’s myocardium after MSCs, MSCs-CM, and SM transplantation were analyzed by RT-PCR. Compared to the DOX group the levels of Bcl-2 were significantly highest in the MSCs and MSCs-CM group (p<0.01; Fig 8), however there was no significant difference between Bax expression levels in these groups. The SM (DMEM) group had the relative expression levels of apoptotic genes (Bcl2 and Bax) nearest to the control group. Details of these expressions are shown in fig 9.

##### Assay of HGF and IGF-1 for evaluating paracrine function of MSCs

**Fig 10:**
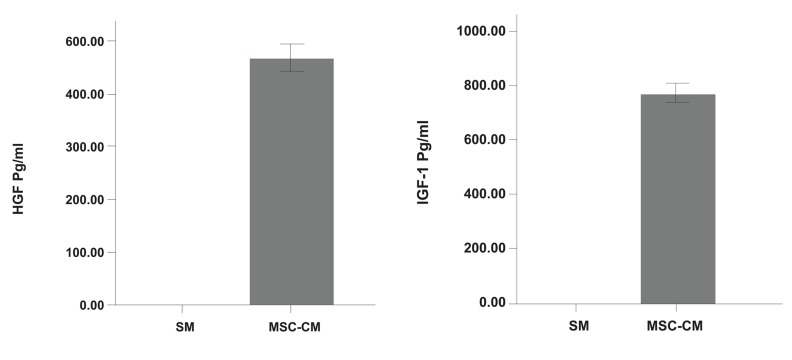
MSCs-conditioned medium (MSCs-CM) contains HGF and IGF-1. There are significant differences (p<0.01) in both HGF and IGF-1 between MSCs-CM and standard medium (SM; DMEM).

To investigate whether MSCs secreted anti-fibrotic and anti-apoptotic factors in CM, IGF-1 and HGF levels were measured by ELISA. It was noted that MSCs secreted large amounts of IGF-1 and HGF (p<0.01). Production of HGF (pg/ml)and IGF-1 (pg/ml) are shown in Fig 10.

## Discussion

In this article we provided evidence that transplantation of MSCs attenuated cardiac fibrosis associated with DOX, increased Bcl2 expression in the-myocardium treated with DOX and exerted anti-fibrotic and anti-apoptotic effects on the myocardium, probably by acting in a paracrine manner.

The results of this study showed that DOX injections significantly (p<0.01) increased interstitial fibrosis, and caused decreased Bcl2 and increased Bax expression levels compared to the control group. These results supported the findings of previous studies where Chen et al. showed that DOX treatment increased myocardial interstitial fibrosis, which was also accompanied by a decrease in Bcl-2 and an increase in Bax expression levels in the rat heart (25). The results of this study also showed that intravenous injection of MSCs decreased cardiac fibrosis associated with DOX administration. Nagaya et al. also showed that intravenous injection of MSCs decreased the fibrosis zone in a rat model of acute myocardial infarction (26). In addition, Ohnishi et al. demonstrated that intravenous injection of MSCs attenuated myocardial injury, dysfunction, and fibrosis in a rat model of acute myocarditis (27). This study revealed that MSCs increased Bcl2 expression levels in the myocardium compared with the DOX group. Previous studies also showed that MSCs reduced cardiomyocyte apoptosis and increased the Bcl-2 to Bax protein ratio in myocardial infarction (28).

Members of the Bcl-2 family are key regulators of apoptosis (29); these proteins control the unity of the outer membrane mitochondria (30). The Bcl-2 protein family consists of both anti-apoptotic members, which are comprised of Bcl-2 and Bcl-xl (which inhibits cytochrome c release into the cytosol and prevents apoptosis), and pro-apoptotic members that are comprised of Bid, Bax, and Bad (which cause the release of cytochrome c by promoting mitochondrial pore opening) (30, 31). These results have revealed the anti-fibrotic and anti-apoptotic effects of MSCs on DOX-induced fibrosis.

In the next step, we aimed to investigate mechanisms that mediated the anti-fibrotic and anti-apoptotic effects of MSCs. In this study we showed that intravenous injection of CM also decreased myocardial interstitial fibrosis and increased Bcl-2 expression levels compared to the DOX group. Our investigation of CM also revealed that MSCs secreted large amounts of HGF and IGF-1. HGF is a growth factor that has been shown to exert anti-fibrotic and anti-apoptotic effects (25, 33). Previous studies have shown that HGF levels in the myocardium of cardiomyopathic rats were significantly lower compared with those in normal hearts (32). HGF gene transfer into the myocardium of these rats significantly decreased the size of the fibrotic area (34). IGF-1 is a growth hormone that plays an important role in myocardial and skeletal muscle growth (35, 36).

Administration of IGF-1 after myocardial infarction improves cardiac function through the increase of myocardial growth (37). Anti-fibrotic and anti-apoptotic properties of IGF-1 have been demonstrated in various models of myocardial ischemia (38). Previous studies have also demonstrated that MSC-CM protected cardiomyocytes from hypoxia/reoxygenation-induced apoptosis, prevented the release of cytochrome C from the mitochondria, and decreased caspase-3 activation, which suggested that MSCs CM protected cardiomyocytes by interfering with a mitochondria-mediated apoptotic pathway in a paracrine manner (39).

According to our observations in this study and other references, we suggest that MSC-derived HGF and IGF-1 may participate in the anti-apoptotic and anti-fibrotic effect of MSCs on the DOX model of heart fibrosis. While MSCs can secrete several cytokines, further studies will be required to detect additional cytokines that may also mediate the anti-fibrotic and anti-apoptotic effects of MSCs.

## Conclusion

The results of this study indicate that MSC transplantation can attenuate myocardial fibrosis and increase Bcl-2 expression levels following DOX injection. The beneficial effects of MSCs transplantation may be, at least in part, due to paracrine action and HGF and IGF-1 secretion.
